# Fibrinogen-like protein 2-complement C3 interaction exacerbates tubular inflammation in acute kidney injury by elevating complement C3a levels

**DOI:** 10.1080/0886022X.2026.2701621

**Published:** 2026-07-20

**Authors:** Wenli Wan, Qun Yang, Jiayue Li, Shun Wu, Xueqing Hu, Zhenmin Ruan, Rui Wang, Zhaoyong Zhang, Xin Geng, YuXin Zhou, Qian Lu, Hongqi Ren

**Affiliations:** a Department of Nephrology, Huaihai Hospital of Xuzhou Medical University, Xuzhou, China; b School of Pharmacy, Xuzhou Medical University, Xuzhou, China

**Keywords:** acute kidney injury, fibrinogen-like protein 2, inflammation, renal tubular epithelial cells, complement C3

## Abstract

Renal tubular epithelial cells are among the earliest renal parenchymal cells to be injured in the context of acute kidney injury (AKI). Numerous studies have confirmed that fibrinogen-like protein 2 (FGL2) can regulate the occurrence and development of inflammation during disease progression. We found that FGL2 expression is elevated under AKI conditions. However, the role of FGL2 in AKI remains unclear. To elucidate the role of FGL2 in AKI, we employed lentiviral and adeno-associated virus for transfection in cellular and murine models. Furthermore, by leveraging datasets from the gene expression omnibus and gene set enrichment analysis databases, we identified the inflammation-related genes in AKI and predicted their interaction with FGL2. Both *in vivo* and *in vitro* studies showed that overexpression of FGL2 markedly increased complement C3a (C3a) levels and exacerbated inflammation and injury. In contrast, knockdown of FGL2 resulted in a marked decline in C3a levels, which not only conferred a substantial protective effect against hypoxia/reoxygenation-induced injury in tubular cells but also effectively alleviated kidney injury in mice. At the molecular level, FGL2 interacts with complement C3, leading to elevated C3a production. This stimulates the inflammatory response of renal tubular epithelial cells, thereby inducing tubular damage. Targeting FGL2 may hold potential prevention and treatment strategy for tubular injury in AKI.

## LAY SUMMARY

AKI is a sudden loss of kidney function, often driven by inflammation. In this study, we investigated a protein called FGL2, which is involved in immune responses. We found that FGL2 levels increase in the kidney during AKI. FGL2 binds to complement C3, leading to excessive production of C3a. This process exacerbates damage to the kidney’s tubules. Our findings suggest that FGL2-C3 interaction is a key driver of kidney inflammation in AKI, and targeting this interaction may offer a new therapeutic strategy.

## KEY MESSAGES

What is known: Inflammation is a key driver of kidney damage in acute kidney injury, but the molecular mechanisms remain incompletely understood.

This study adds: Fibrinogen-like protein 2 binds to complement C3 in the kidney, leading to excessive C3a production and exacerbated tubular inflammation during acute kidney injury.

Potential impact: Targeting the fibrinogen-like protein 2-C3 interaction may represent a novel therapeutic approach for acute kidney injury.

## Introduction

AKI is a common and critical clinical condition, characterized by a rapid decline in renal function within a short timeframe [1]. The core pathological mechanism of AKI involves primary severe damage, leading to apoptosis or necrosis of renal tubular epithelial cells. This cellular injury subsequently triggers intense inflammatory responses, ultimately exacerbating renal dysfunction [2]. If such a damage is not repaired in a timely and effective manner, it can lead to irreversible injury to renal parenchymal cells and nephron structures, resulting in sustained renal dysfunction and ultimately progressing to chronic kidney disease [3,4]. During the acute cellular injury phase of AKI, the inflammatory response serves as a key pathogenic driver [5,6]. Inflammation-mediated acute tubular necrosis exacerbates AKI progression [7,8]. Accumulating evidence demonstrates that injured renal tubules propel the initiation and perpetuation of post-AKI inflammation *via* overexpression of pattern recognition receptors. Pattern recognition receptors orchestrate the recruitment and activation of inflammatory cells, culminating in progressive renal dysfunction [9,10]. Fibrinogen-like protein 2 (FGL2), a member of the fibrinogen-related protein superfamily, directly catalyzes the conversion of prothrombin to thrombin, thereby promoting coagulation [11]. Additionally, FGL2 serves as a key immunomodulatory molecule and is involved in the pathological processes of various immune-inflammatory diseases [12,13]. The studies have revealed that FGL2 participates in the maintenance of immune homeostasis and the regulation of inflammatory responses through multiple mechanisms [14]. FGL2 exhibits broad constitutive expression across a variety of organs and tissues, including the kidney, liver, and spleen [15,16]. Moreover, its expression can be induced in various cell types, such as endothelial cells, macrophages, and lymphocytes [17,18]. Recent studies have demonstrated that FGL2 plays an important role in unilateral ureteral obstruction-induced renal fibrosis [19]. However, the mechanism of FGL2 in AKI associated tubular pathology remains unclear.

Through integrated bioinformatics analysis, we predicted a putative interaction between complement C3 (C3) and FGL2. This was further supported by Co-IP assays, which confirmed the protein-protein interaction between FGL2 and C3. C3 plays a central role in the classical, alternative, and lectin pathways of the complement system. Its cleavage products are critically involved in immune regulation and inflammatory responses [20]. Studies have indicated that the complement system contributes to excessive inflammation in AKI [21]. For instance, the C3a/C3aR pathway can promote the formation of neutrophil extracellular traps, thereby exacerbating renal dysfunction [22,23]. Additionally, studies have found that C3 is aberrantly activated in AKI, suggesting its potential as a therapeutic target for this condition.

This study aimed to explore if FGL2 promotes C3 activation and complement C3a (C3a) generation, thereby amplifying inflammation and worsening tubular injury. By revealing a new mechanism of FGL2-driven tubular inflammation, this study provides fresh insight into the pathogenesis of AKI.

## Methods and materials

### Clinical research

This study was carried out in strict compliance with the ethical standards established by the Ethics Committee of Huaihai Hospital of Xuzhou Medical University, and has obtained approval from the committee (Approval No.: LL-2022YX02). The study was conducted with the written informed consent of all participants, who agreed to sample collection for the specified analyses.

We recruited healthy controls from the Health Examination Center and patients diagnosed with AKI from the Nephrology Department of the Affiliated Huaihai Hospital of Xuzhou Medical University during the period from October 2022 to June 2025. Residual serum samples, which were obtained after routine clinical testing, were collected for subsequent analysis. The expression levels of FGL2 in patient serum were quantified using enzyme-linked immunosorbent assay kits (Jianglai, JL33587). Detailed baseline characteristics of the participants are presented in Supplementary Table S1. The inclusion and exclusion criteria for the study subjects can be found in the Supplementary Materials.

### Cell culture

The Human Kidney-2 (HK-2) cells were procured from Shanghai Lianmai Biotechnology Co., Ltd (Shanghai, China). The cells were cultured in low-glucose Dulbecco’s Modified Eagle Medium (Gibco, C11885500bt) supplemented with 10% fetal bovine serum (Beyotime, C0226) and 1% penicillin-streptomycin (Gibco, 15140122) under standard adherent culture conditions at 37 °C in a 5% CO_2_ incubator. An *in vitro* hypoxia model was established by treating cells with CoCl_2_ (Sigma, 202185). Following 24 h incubation under hypoxic conditions, the cells were transferred to complete medium without CoCl_2_ and cultured under reoxygenation conditions for 6 h, after which cellular samples were collected for subsequent assays. Detailed information regarding the cell experiment can be found in the Supplementary Materials.

### Animal experiment

Eight-week-old male C57BL/6J mice of specific pathogen-free grade were purchased from Changzhou Cavens Laboratory Animal Co., Ltd (Changzhou, China). These animals were transported under sterile conditions to the barrier facility at the Experimental Animal Center of Xuzhou Medical University. Throughout the study, environmental conditions were strictly controlled: the temperature was maintained at 24 ± 1 °C, relative humidity at 50 ± 10%, and a 12 h/12 h light-dark cycle was in place. All mice had free access to food and water. The animal experiments were approved by the Animal Ethics Committee of Xuzhou Medical University (Approval No.: 202501T003). At the experimental endpoint, mice were euthanized by CO_2_ inhalation using 100% compressed gas delivered at a flow rate of ∼40% chamber volume/min, followed by cervical dislocation to confirm death after respiratory and cardiac arrest. The specific details of the animal experiment are provided in the Supplementary Materials.

### Real-time polymerase chain reaction

Total RNA was isolated from renal tissues and HK-2 cells utilizing TRIzol reagent. Following this, the isolated RNA was reverse-transcribed into complementary DNA employing a reverse transcription kit (Vazyme, R233-01) supplied by Vazyme Biotech. Subsequently, qPCR analysis was carried out on a LightCycler^®^ 480 Instrument II (Shanghai, China). The reaction substrates were designed by Shanghai Generay Biotech Co., Ltd (Shanghai, China). All primers used were listed in Supplementary Table S2.

### Western blotting

Western blotting analysis was conducted to detect proteins that had been extracted from the mouse renal cortex and HK-2 cells. For this analysis, the following primary antibodies were employed: C3 (Abcam, Ab181147), FGL2 (Proteintech, 11827-1-AP), IL-6 (Proteintech, 21865-1-AP), KIM-1 (Proteintech, 83221-2-RR), NGAL (Proteintech, 30700-1-AP), TNF-α (Proteintech, 29652-1-AP), and β-actin (Proteintech, 60008-1-Ig). All antibodies used were listed in Supplementary Table S3.

### Biochemical levels assessment

The levels of serum urea nitrogen (BUN) (Jiancheng, C013-2-1), serum creatinine (SCr) (Jiancheng, C011-2-1), and superoxide dismutase (SOD) (Jiancheng, A001-1) were determined using assay kits sourced from Nanjing Jiancheng Bioengineering Institute (Nanjing, China). Meanwhile, the serum malondialdehyde (MDA) (Beyotime, S0131M) content was measured with a kit obtained from Shanghai Beyotime Biotechnology.

### Enzyme-linked immunosorbent assay

The levels of FGL2 (Jianglai, JL33587) in clinical serum samples, as well as the levels of C3a (Jianglai, JL10879) (Meimian, ml105132), IL-1β (Jianglai, JL18442) (Meimian, ml028592), TNF-α (Huabodeyi, HBDY-60770H2) and IL-6 (Huabodeyi, HBDY-50441H2) in cell supernatants and mouse sera, were all detected using the enzyme-linked immunosorbent assay. The specific experimental procedures were carried out in strict accordance with the instructions provided in the corresponding reagent kits.

### Immunofluorescence analysis

Paraffin-embedded renal tissue sections were first deparaffinized and rehydrated to water, followed by three rounds of washing with phosphate-buffered saline. Antigen retrieval was then carried out by treating the sections with 0.4% pepsin (Leagene, IH0308) at 37 °C for 30 min. Next, the tissue sections were blocked with fast-acting sealant (NCM Biotech, P30500) for 30 min. Subsequently, the sections were incubated overnight at 4 °C with primary antibodies. Then, corresponding species-specific fluorescent secondary antibodies were applied for detection (Donkey Anti-Rabbit IgG H&L preadsorbed, Abcam, ab96921) (Donkey Anti-Mouse IgG H&L preadsorbed, Abcam, ab98794). The cell nuclei were counterstained with 4′,6-diamidino-2-phenylindole (Beyotime, P0131). Finally, fluorescence intensity images were visualized and captured using an Olympus BX43 fluorescence microscope. All antibodies used were listed in Supplementary Table S3.

### Renal morphological assessment

Paraffin-embedded renal tissue sections with a thickness of 3 μm were prepared. Subsequently, these sections were stained using Hematoxylin and eosin (H&E) (Servicebio, G1005), Periodic acid-Schiff (PAS) (Servicebio, G1008), and Masson staining kits (Servicebio, G1006) to facilitate the observation of renal histomorphology. Staining kit from Wuhan Servicebio Technology Co., Ltd (Wuhan, China) was used to stain renal paraffin sections, the procedure was carried out strictly in accordance with the manufacturer’s protocol. All microscopic images were randomly acquired using an Olympus BX53 microscope equipped with appropriate imaging software for subsequent analysis. This grading scale (0 = no injury; 1 = <10%; 2 = 10–25%; 3 = 26–50%; 4 = >50% of affected renal tubules) was used to evaluate tubular necrosis, cast formation, tubular dilatation, brush border loss, and vacuolar degeneration. All slides were scored independently by two observers blinded to the experimental groups to ensure objective assessment.

### Data collection and processing

Download the GSE87024 and GSE39766 datasets from the gene expression omnibus (GEO) database (http://www.ncbi.nlm.nih.gov/geo/). Used the R package “limma” to perform normalization and batch effect correction on the merged dataset to eliminate non-biological technical variations. Subsequently, employed the “DESeq2” package for differential expression analysis. Meanwhile, searched the gene set enrichment analysis (GSEA) database for gene sets related to acute inflammation. First took the intersection of DEG from the two datasets. Then, took the intersection of this gene set with the aforementioned acute inflammation related gene sets to ultimately screen out core genes.

### Molecular docking

Starting with the gene name, retrieved the protein sequence from UniProt, inputted it into SWISS-MODEL to obtain the optimal protein structure, and then used the resulting PDB file for protein-protein docking on the ZDOCK server (https://zdock.wenglab.org/). All data were listed in Supplementary Table S4.

### Co-immunoprecipitation

HK-2 cells were maintained under either normal culture conditions or exposed to hypoxia for a duration of 24 h, which was then succeeded by 6 h of reoxygenation. Subsequently, cells from both the normal culture group and the H/R group were harvested for Co-IP experiments. Detailed experimental procedures are available in the Supplementary Materials. All antibodies used were listed in Supplementary Table S3.

### Statistical analysis

The data are displayed in the format of Mean ± standard error of the mean (SEM). Statistical analyses were conducted utilizing GraphPad Prism version 8.0 (GraphPad Software Inc., San Diego, CA). The differences among groups were evaluated through one-way analysis of variance, followed by Tukey’s multiple comparisons test. A *p*-value of less than 0.05 was deemed to indicate statistical significance.

## Results

### FGL2 expression is upregulated in AKI

**Figure 1.**
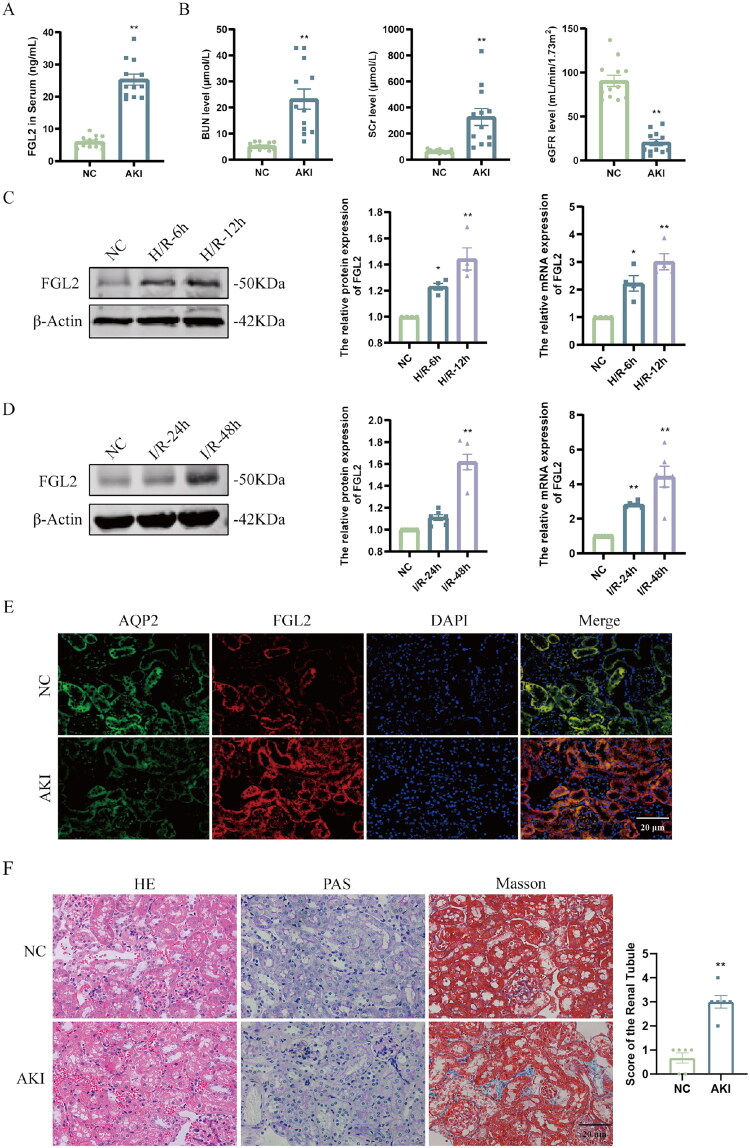
FGL2 expression is upregulated in AKI. (A) Serum FGL2 levels in normal individuals and patients with AKI, *n* = 12; (B) BUN, SCr, eGFR levels in normal individuals and patients with AKI, *n* = 12; (C) FGL2 protein expression and statistical results in renal tubular epithelial cells, FGL2 mRNA expression levels in renal tubular epithelial cells, *n* = 4; (D) FGL2 protein expression and statistical results in mouse kidneys, FGL2 mRNA expression in mouse kidneys, *n* = 6; (E) Fluorescence microscopy images of FGL2 (red) and AQP2 (green) in the kidney of NC and AKI mice, scale bar = 20 μm; (F) H&E, PAS, Masson staining, scale bar = 20 μm. *Note.* The normal control group (NC); the acute kidney injury group (AKI); the renal ischemia followed by 24 h reperfusion group (I/R-24 h); the renal ischemia followed by 48 h reperfusion group (I/R-48 h); the hypoxia followed by 6 h reoxygenation group (H/R-6 h); the hypoxia followed by 12 h reoxygenation group (H/R-12 h). Data were represented as Mean ± SEM. *p < 0.05; **p < 0.01, compared with the NC group.

We measured the expression levels of FGL2 in the serum of healthy individuals and AKI patients, the results showed that serum FGL2 levels were significantly elevated in AKI patients compared to healthy individuals (Figure 1(A)). The key renal function indicators in patients with AKI exhibited significant abnormalities (Figure 1(B)) and FGL2 levels showed significant correlations with eGFR, BUN, and SCr (Supplementary Figure S4A). To further validate the expression of FGL2 in AKI, we established an *in vitro* AKI model in HK-2 cells. WB and qPCR results showed that FGL2 expression was also increased in HK-2 cells after H/R treatment (Figure 1(C)). Similarly, in the *in vivo* model, WB and qPCR results demonstrated that the expression of FGL2 in the kidneys of mice was significantly upregulated at both 24 h and 48 h after ischemia/reperfusion (I/R) (Figure 1(D)). Immunofluorescence results showed that the expression of FGL2 in mouse kidneys colocalized with AQP2 in renal tubules (Figure 1(E)). Histopathological analysis revealed marked vacuolar degeneration in the renal tubule regions of the AKI model group (as shown by HE and PAS staining), Masson staining indicated collagen fiber deposition in the kidneys of mice model. Semi-quantitative analysis showed that the injury score in the AKI group was significantly higher than that in the NC group (Figure 1(F)). The above results supporting the conclusion that FGL2 expression is upregulated in AKI and is related to renal function.

### Overexpression of FGL2 induces renal inflammation in mice

**Figure 2.**
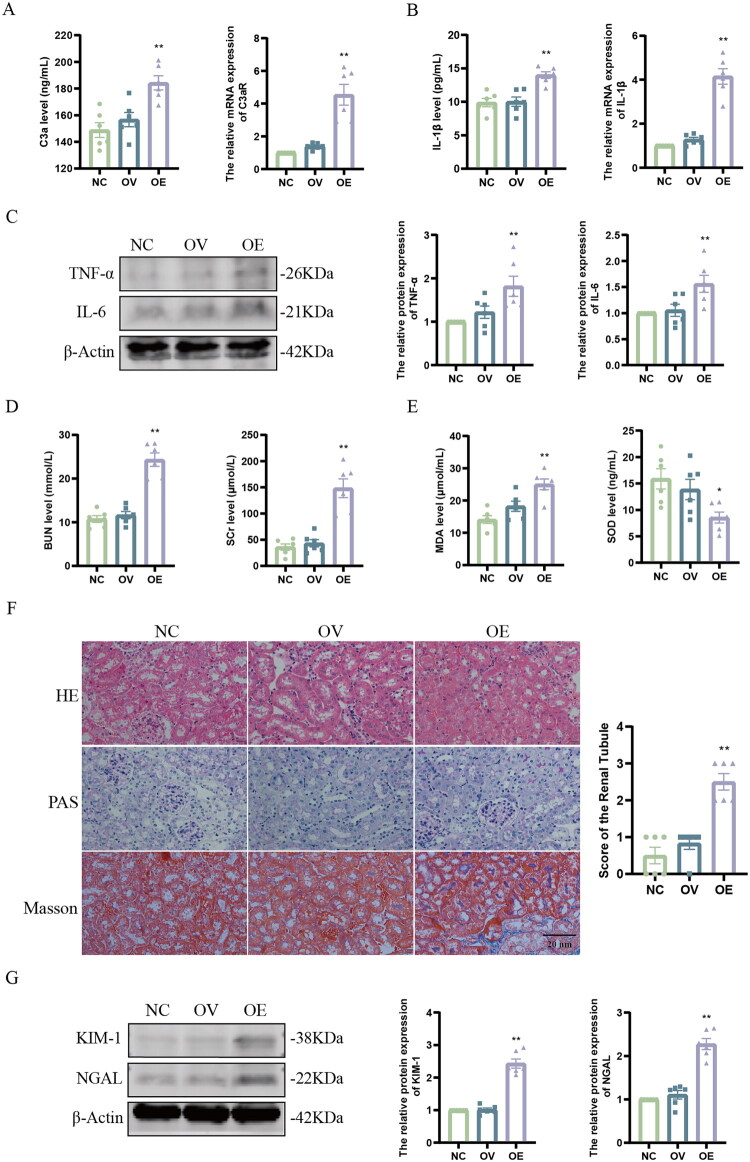
Overexpression of FGL2 induces renal inflammation in mice. (A) C3a levels in mouse serum, C3aR mRNA expression levels in mouse kidneys, *n* = 6; (B) IL-1β levels in mouse serum, IL-1β mRNA expression levels in mouse kidneys, *n* = 6; (C) The expression levels of inflammation-related proteins TNF-α and IL-6 in the kidneys of mice from each group, *n* = 6; (D) BUN and SCr levels in mouse serum, *n* = 6; (E) MDA and SOD levels in mouse serum, *n* = 6; (F) H&E, PAS, and Masson staining, scale bar = 20 μm; (G) The expression levels of KIM-1 and NGAL in the kidneys of mice from each group, *n* = 6. *Note.* The normal control group (NC); the empty vector group (OV); the FGL2 overexpression group (OE). Data were represented as Mean ± SEM. *p < 0.05; **p < 0.01, compared with the NC group.

To validate the functional role of FGL2 in the kidneys of mice, we established an animal model with FGL2 overexpression (Supplementary Figure S1). The immunofluorescence co-staining results indicate that FGL2 is primarily expressed in renal tubular epithelial cells (Supplementary Figure S3). ELISA detection of the complement activation fragment C3a and qPCR analysis of its receptor C3aR showed that both were significantly increased in the kidneys of the overexpression group (Figure 2(A)). Meanwhile, the expression of IL-1β was also found to be increased upon detection (Figure 2(B)). Further WB analysis demonstrated markedly upregulated protein expression of TNF-α and IL-6 in renal tissues of the overexpression group (Figure 2(C)). Subsequent biochemical analysis revealed significantly elevated levels of renal function markers (BUN, SCr, Figure 2(D)) and oxidative stress (MDA, SOD, Figure 2(E)) in the overexpression group. Histopathological examination revealed prominent vacuolar degeneration in the renal tubules of the overexpression group (HE and PAS staining). Masson staining indicated that, compared with the control group, collagen fiber deposition in the kidney tissue of mice overexpressing FGL2 was increased. Semi-quantitative analysis showed that the injury score in the OE group was significantly higher than that in the NC group (Figure 2(F)). Additionally, WB analysis showed significantly elevated expression of tubular injury markers including KIM-1 and NGAL (Figure 2(G)), confirming that FGL2 overexpression caused significant renal tubular injury.

### Overexpression of FGL2 induces inflammation in HK-2 cells

**Figure 3.**
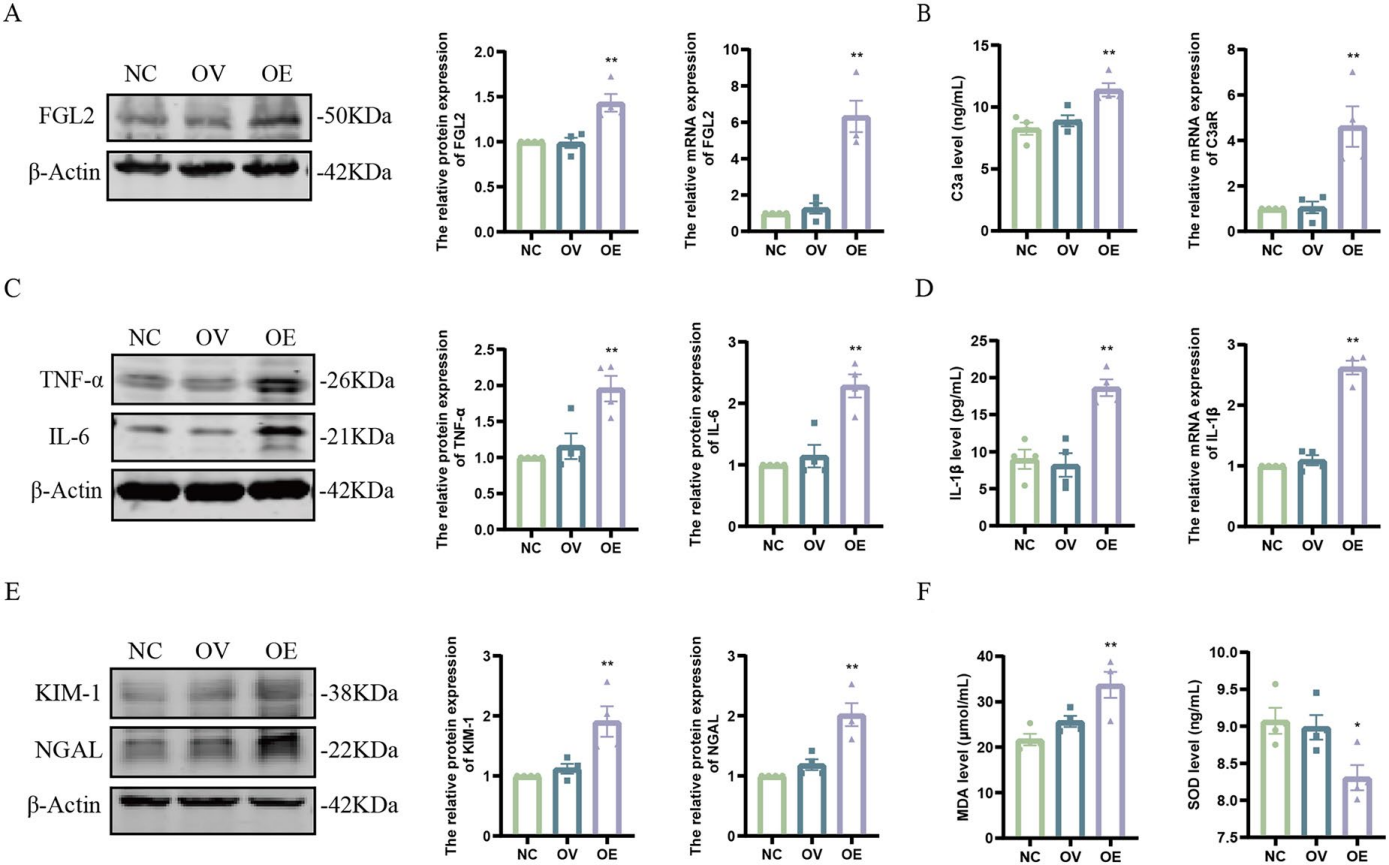
Overexpression of FGL2 induces inflammation in HK-2 cells. (A) FGL2 protein expression levels in renal tubular epithelial cells, FGL2 mRNA expression levels in renal tubular epithelial cells, *n* = 4; (B) The levels of C3a in cells from each group, the C3aR mRNA levels in cells from each group, *n* = 4; (C) The protein expression of TNF-α and IL-6 in cells from each group, *n* = 4; (D) The levels of IL-1β in cells from each group, the IL-1β mRNA levels in cells from each group, *n* = 4; (E) The expression of KIM-1 and NGAL in cells from each group, *n* = 4; (F) MDA and SOD levels in cells from each group, *n* = 4. *Note.* The normal control group (NC); the empty vector group (OV); the FGL2 overexpression group (OE). Data were represented as Mean ± SEM. *p < 0.05; **p < 0.01, compared with the NC group.

To establish an FGL2-overexpressing cell model, we infected HK-2 cells with a lentiviral vector. The results from WB and qPCR show that after transfection with an overexpressed lentivirus, the expression level of FGL2 in HK-2 cells has increased (Figure 3(A)). Subsequently, we observed that both the level of C3a and the mRNA expression of its receptor C3aR were significantly elevated in the overexpression group (Figure 3(B)). WB analysis further showed increase in the protein expression of TNF-α and IL-6 in the overexpression group (Figure 3(C)). Moreover, ELISA analysis and qPCR analysis both revealed upregulation of IL-1β in the overexpression group (Figure 3(D)). Additionally, WB detection showed a significant upregulation of KIM-1 and NGAL in the overexpression group (Figure 3(E)). In the cell lysates of the overexpression group, the MDA level was elevated, while the SOD level was decreased (Figure 3(F)), suggesting that FGL2 overexpression induced injury in these cells.

### Knockdown of FGL2 alleviates renal inflammation in mice with AKI

**Figure 4.**
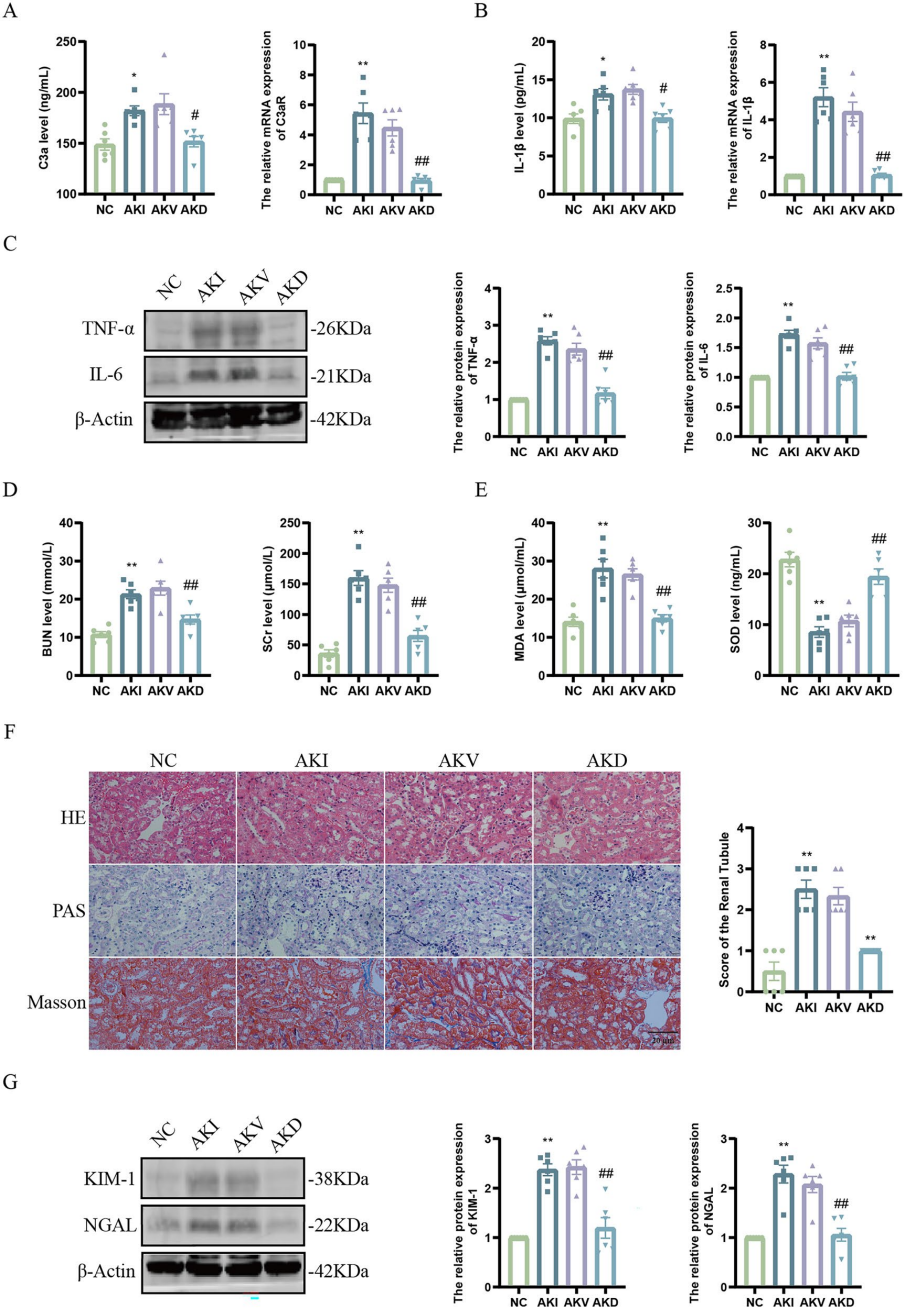
Knockdown of FGL2 alleviates renal inflammation in mice with AKI. (A) The C3a levels in the serum of mice from each group, the C3aR mRNA expression levels in the kidneys of mice from each group, *n* = 6; (B) The IL-1β levels in the serum of mice from each group, IL-1β mRNA expression levels in the kidneys of mice from each group, *n* = 6; (C) The expression levels of TNF-α and IL-6 in the kidneys of mice from each group, *n* = 6; (D) BUN and SCr levels in the serum of mice from each group, *n* = 6; (E) MDA and SOD levels in the serum of mice from each group, *n* = 6; (F) H&E, PAS, Masson staining, scale bar = 20 μm; (G) The expression levels of KIM-1 and NGAL in the kidneys of mice from each group, *n* = 6. *Note.* The normal control group (NC); the AKI group (AKI); the AKI + empty vector group (AKV); the AKI + FGL2 knockdown group (AKD). Data were represented as Mean ± SEM. *p < 0.05; **p < 0.01, compared with the NC group. ^#^*p* < 0.05, ^##^*p* < 0.01, compared with the AKI group.

To validate the functional role of FGL2 in the kidneys of mice with AKI, we established an AKI animal model with FGL2 knockdown (Supplementary Figure S2). ELISA or qPCR analyses showed that, compared with the normal group, both C3a and C3aR were significantly elevated in the AKI group; however, their levels were reduced in the AKD group compared with the AKI group (Figure 4(A)). The same pattern was observed in the ELISA or qPCR detection results of IL-1β (Figure 4(B)). Further WB analysis demonstrated that the protein expression of pro-inflammatory factors TNF-α and IL-6 was significantly increased in the AKI group after I/R, whereas their expression was markedly reduced in the AKD group compared with the AKI group (Figure 4(C)). Compared with the normal group, the AKI group had significantly elevated levels of renal function indicators (BUN, SCr, Figure 4(D)) and the level of oxidative stress (MDA, SOD, Figure 4(E)). In contrast, the levels of renal function indicators and oxidative stress were significantly lower in the AKD group than in the AKI group. Histopathological analysis demonstrated that compared with the AKI group, the AKD group exhibited less renal structural damage and pathological changes. Semi-quantitative analysis showed that the injury score in the AKD group was significantly lower than that in the AKI group (Figure 4(F)). WB analysis showed that the expression of KIM-1 and NGAL was significantly elevated in the AKI group but reduced in the AKD group (Figure 4(G)), suggesting that FGL2 knockdown effectively attenuated renal tubular injury.

### Knockdown of FGL2 alleviates H/R-induced inflammation in HK-2 cells

**Figure 5.**
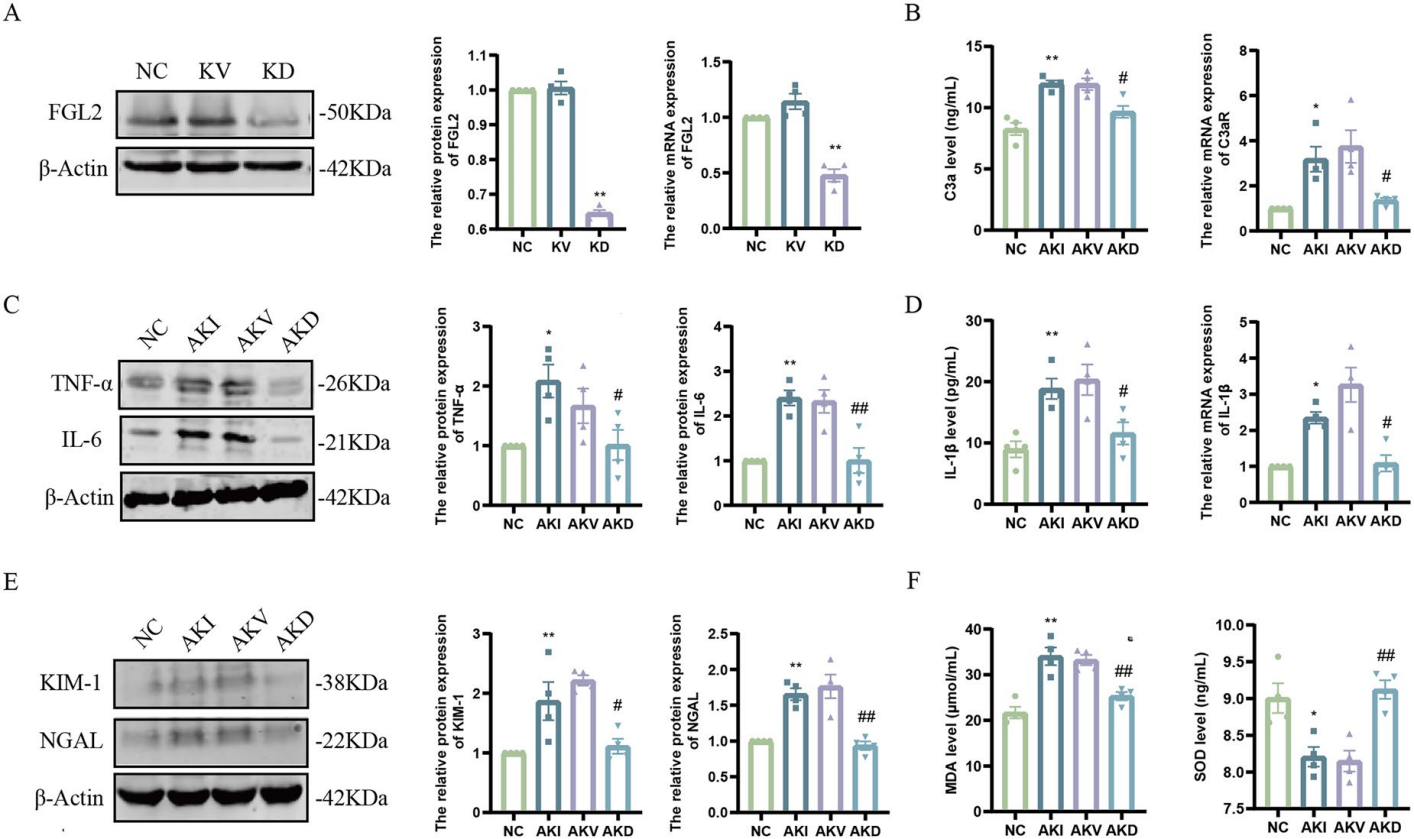
Knockdown of FGL2 alleviates H/R-induced inflammation in HK-2 cells. (A) The FGL2 protein and mRNA expression levels in renal tubular epithelial cells, *n* = 4; (B) The levels of C3a in cells from each group, the C3aR mRNA levels in cells from each group, *n* = 4; (C) The protein expression of inflammation-related proteins TNF-α and IL-6 in cells from each group, *n* = 4; (D) The protein levels of IL-1β in cells from each group, the IL-1β mRNA levels in cells from each group, *n* = 4; (E) The expression of KIM-1 and NGAL in cells from each group, *n* = 4; (F) MDA and SOD levels in cells from each group, *n* = 4. *Note.* The normal control group (NC); the AKI group (AKI); the AKI + empty vector group (AKV); and the AKI + FGL2 knockdown group (AKD). Data were represented as Mean ± SEM. *p < 0.05; **p < 0.01, compared with the NC group. ^#^*p* < 0.05, ^##^*p* < 0.01, compared with the AKI group.

FGL2-knockdown cell model was established in HK-2 cells using lentiviral infection. WB and qPCR results showed that the expression in the FGL2 knockdown group was significantly reduced (Figure 5(A)). Subsequently, analysis revealed that the level of C3a and the mRNA expression of its receptor C3aR were increased in the AKI group, whereas both levels in the AKD group were decreased (Figure 5(B)). Additionally, WB analysis revealed that the protein expression of TNF-α and IL-6 was significantly elevated in the AKI group after hypoxia/reoxygenation. ELISA and qPCR assays further confirmed a marked increase in IL-1β in the AKI group. In contrast, all these inflammatory factors were reduced in the AKD group (Figure 5(C, D)). Finally, WB results showed that the expression of KIM-1 and NGAL was upregulated in the AKI group, whereas their expression was markedly decreased in the AKD group compared with the AKI group (Figure 5(E)). The level of MDA was elevated in the AKI group, while the activity of SOD was decreased, whereas the AKD group demonstrated a notable improvement when compared to the AKI group (Figure 5(F)). Confirmed that FGL2 knockdown provides protection against H/R-induced injury in renal tubular epithelial cells.

### C3 is the key molecule that FGL2 uses to drive AKI inflammation

**Figure 6.**
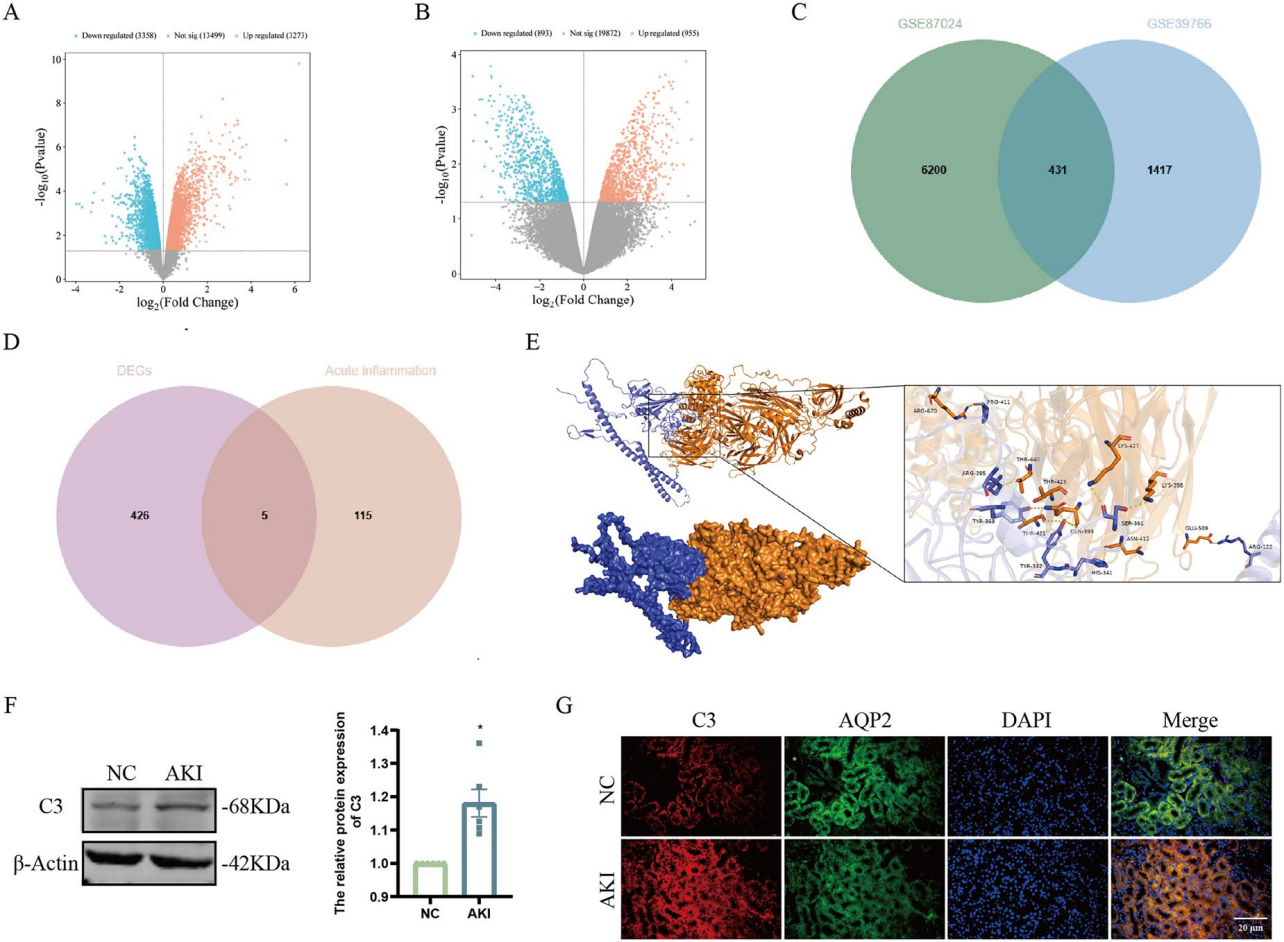
C3 is the key molecule that FGL2 uses to drive AKI inflammation. (A) DEG in GSE87024; (B) DEG in GSE39766; (C) Differentially co-expressed genes between GSE87024 and GSE39766; (D) Core targets in acute inflammation during AKI; (E) Schematic diagram of the binding mode between FGL2 and C3; (F) The C3 protein expression levels in the kidneys of mice, *n* = 6; (G) Fluorescence microscopy images of C3 (red) and AQP2 (green) in the kidneys of NC and AKI mice, scale bar = 20 μm. *Note.* The normal control group (NC); the acute kidney injury group (AKI). Data were represented as Mean ± SEM. *p < 0.05; **p < 0.01, compared with the NC group.

To identify key inflammatory factors in AKI, we conducted an integrated analysis of the GSE87024 and GSE39766 datasets. The GSE87024 dataset revealed 3273 significantly upregulated genes and 3358 significantly downregulated genes (Figure 6(A)), and the GSE39766 dataset showed 955 upregulated genes and 893 downregulated genes (Figure 6(B)). Intersecting these DEG identified 431 common DEG that were consistently altered in AKI (Figure 6(C)). Further intersection with acute inflammation-related gene sets pinpointed five core genes (Figure 6(D)). To investigate potential interactions between FGL2 and these core genes, molecular docking simulations were performed. The results indicated a binding interaction between FGL2 and C3 (Figure 6(E)), suggesting that the FGL2-C3 protein interaction may play a critical role in the inflammatory process of AKI. To validate these predictions, we measured C3 expression in renal tissues of mice using WB. The results showed that the AKI group had significantly increased C3 protein levels (Figure 6(F)). Immunofluorescence experiments showed that C3 is expressed in renal tubules (Figure 6(G)).

### FGL2 promotes renal tubular epithelial cell inflammation through interaction with C3

**Figure 7.**
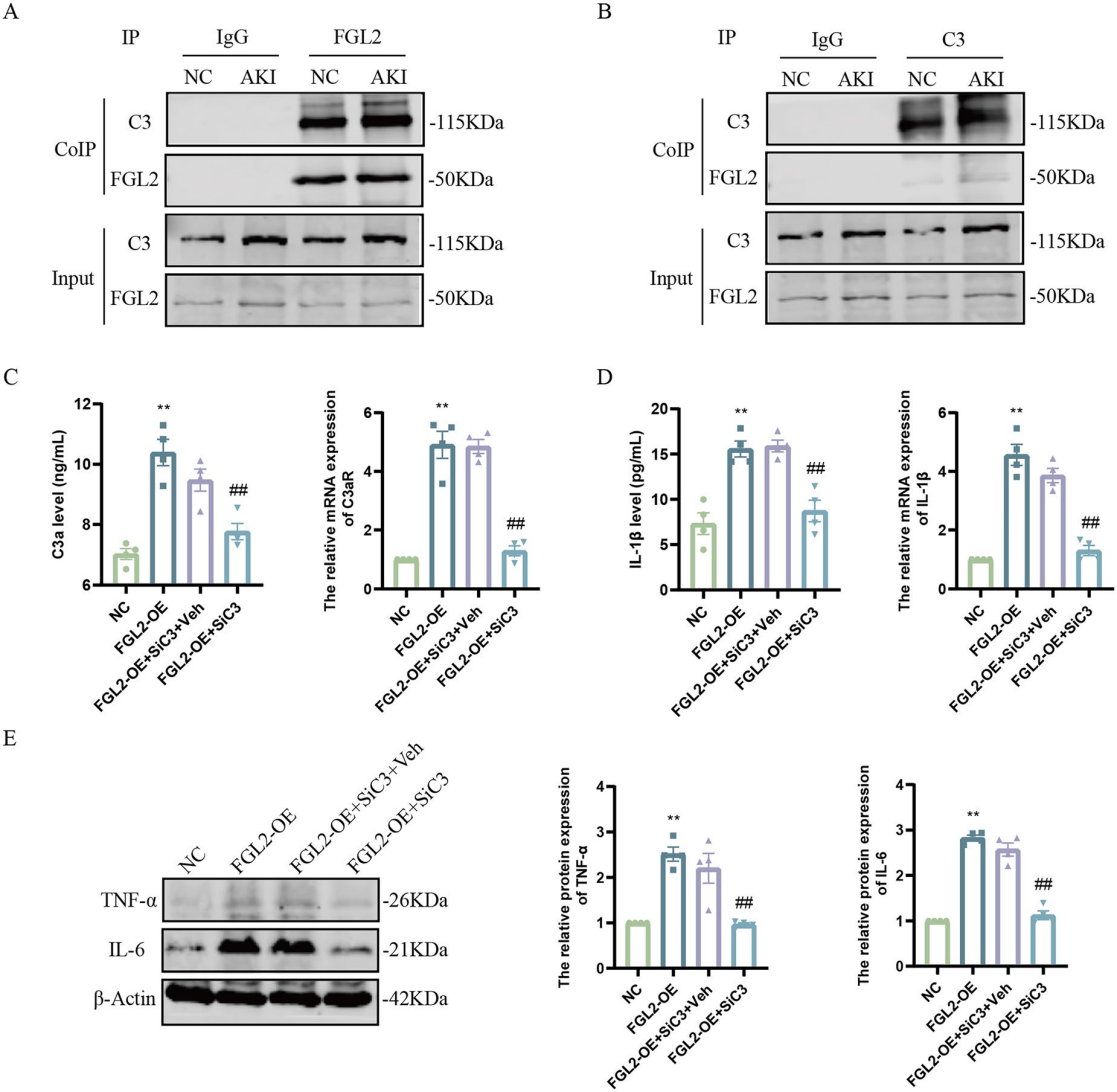
FGL2 promotes renal tubular epithelial cell inflammation through interaction with C3. (A) Co-IP analysis showing the band patterns of FGL2 binding to C3 in HK-2 cells; (B) Co-IP analysis showing the band patterns of C3 binding to FGL2 in HK-2 cells; (C) The C3a protein levels in cells from each group, the C3aR mRNA levels in cells from each group, *n* = 4; (D) The IL-1β protein levels in cells from each group, the IL-1β mRNA levels in cells from each group, *n* = 4; (E) The protein expression of TNF-α and IL-6 in cells from each group, *n* = 4. *Note.* The normal control group (NC); the FGL2 overexpression group (FGL2-OE); the Overexpressing FGL2 silencing C3 empty vector group (FGL2-OE+SiC3 + Veh); the Overexpressing FGL2 silencing C3 group (FGL2-OE+SiC3). Data were represented as Mean ± SEM. *p < 0.05; **p < 0.01, compared with the NC group. ^#^*p* < 0.05, ^##^*p* < 0.01, compared with the AKI group.

To verify whether FGL2 interacts with C3, we performed Co-IP assays. The results showed that FGL2 and C3 exhibit a protein-protein interaction in both normal cells and under AKI conditions (Figure 7(A,B)). Co-IP assays were performed using kidney tissue lysates from I/R-injured mice. The results showed that FGL2 also interacts with C3 *in vivo*, which is consistent with the findings from *in vitro* cellular experiments (Supplementary Figure S4B). Then we transfected C3 shRNA into FGL2-overexpressing HK-2 cells. ELISA detection of C3a and qPCR analysis of C3aR showed that FGL2 overexpression led to increased C3a and C3aR levels; while simultaneous silencing of C3 reduced both (Figure 7(C)). Further analyses, including ELISA and qPCR for IL-1β (Figure 7(D)), and WB for TNF-α and IL-6 (Figure 7(E))—demonstrated that compared with the FGL2 overexpression alone group, the group with both FGL2 overexpression and C3 silencing showed markedly reduced levels of these inflammatory factors. This indicates that C3 silencing significantly alleviates the inflammatory response in renal tubular epithelial cells. Moreover, in HK-2 cells overexpressing FGL2, treatment with the C3aR antagonist SB290157 significantly inhibited the elevation of the inflammatory cytokines IL-6, TNF-α, and IL-1β (Supplementary Figure S4C), indicating that the pro-inflammatory effect of FGL2 is dependent on the C3a-C3aR signaling pathway.

## Discussion

AKI is a common complication among critically ill patients [24]. Repeated renal injuries can activate abnormal renal repair mechanisms, further increasing both the risk of developing CKD [25,26]. One of the key molecular mechanisms of AKI involves persistent inflammatory reactions in the kidney [27]. Investigating the mechanisms underlying the initiation and progression of renal inflammation in AKI is of significant importance for the targeted prevention and treatment. FGL2 knockout has been shown to exert a protective effect in fulminant hepatitis [15]. Similarly, FGL2 can activate thrombin and form a feedback loop with C5a and TNF-α, exacerbating injury [28]. However, the role of FGL2 in AKI remains to be explored. This study provides novel mechanistic insights for this issue. Through clinical, animal, and cell model analyses, this study found that FGL2 levels are significantly elevated in AKI. Immunofluorescence revealed that FGL2 is expressed in mouse renal tubules, suggesting that it may participate in the pathological process of AKI by regulating tubular function.

Based on the aforementioned findings, we established FGL2 overexpression systems both *in vitro* and *in vivo*. The experimental results demonstrated that, compared with the control group, overexpressing FGL2 in mice led to notable renal structural abnormalities and functional decline, accompanied by elevated C3a levels, increased pro-inflammatory cytokine release. Conversely, FGL2 knockdown markedly reduced renal pathology and inflammation. In AKI, complement activation, cytokine release, and tubular injury were observed, all of which were attenuated by FGL2 knockdown. Thus, FGL2 actively drives complement activation and inflammation in AKI, rather than being a passive downstream effect. Knocking down FGL2 breaks this vicious cycle, alleviating inflammation and facilitating tubular repair. The renal tubule is the earliest functional unit affected in AKI [29]. Persistent oxidative stress and inflammation in renal tubular epithelial cells further drive the deterioration of renal function [30,31]. Previous studies have shown that FGL2 knockout suppresses the activation of the complement and coagulation pathways [32]. In HK-2 cells, FGL2 overexpression increased C3a and inflammatory cytokine expression, while FGL2 knockdown reduced C3a levels.

Previous studies have reported abnormal C3 activation in acute kidney injury [33]. Numerous studies have highlighted the critical role of complement activation in AKI [34,35]. For example, C3aR deficiency in mice has been shown to alleviate renal injury [36]. Through bioinformatics analysis, molecular docking, and Co-IP, this study confirmed the interaction between FGL2 and C3. Silencing C3 in FGL2-overexpressing cells reduced C3a and inflammatory factor levels, indicating that FGL2 promotes C3a production and renal tubular inflammation *via* C3 binding. Collectively, these findings establish FGL2 as a key driver of complement-mediated inflammation in AKI. Notably, FGL2 overexpression increased C3aR levels. We found that silencing C3 while overexpressing FGL2 not only reduced C3a levels but also reversed the upregulation of C3aR expression. Furthermore, treatment with C3aR antagonist significantly alleviated FGL2 overexpression-induced inflammation, confirming the necessity of C3aR signaling in FGL2-mediated inflammatory responses.

In summary, this study demonstrates that FGL2 may drive complement activation and renal tubular inflammation in AKI by binding to C3, a mechanism distinct from its role in MASH-related liver fibrosis, where FGL2 acts through C3aR. In MASH, FGL2 interacts with C3aR to promote NETs release from neutrophils, which in turn activates the complement and coagulation systems, exacerbating liver fibrosis. In contrast, in AKI, FGL2 directly binds to C3 to exert its pro-inflammatory effects. Although both mechanisms involve the complement system, they operate at different nodes: in MASH, FGL2 targets C3aR, which lies downstream in the complement cascade, whereas in AKI, FGL2 directly targets C3, acting at a more upstream level of complement activation. Furthermore, the effector cells differ—hepatic neutrophils in MASH versus renal tubular epithelial cells in AKI. This comparison suggests that FGL2 can exert pro-inflammatory functions across different diseases by engaging distinct components of the complement system, highlighting the highly context-dependent nature of its mechanisms.

Several limitations of this exploratory study should be acknowledged. First, although we observed that partial knockdown of FGL2 produced a significant protective effect, suggesting that its function may not be strictly dose-dependent, the precise threshold at which FGL2 transitions from a physiological to a pathogenic role remains undefined. Future studies with graded FGL2 expression levels are warranted to address this issue. Second, while our data demonstrate that FGL2 overexpression elevates C3a levels and that C3 silencing alleviates FGL2-induced injury, the direct causal relationship between FGL2 and C3 cleavage has not been fully established. Specifically, whether FGL2 directly initiates the proteolytic cleavage of complement C3—acting as a C3 convertase-like enzyme or facilitating C3 activation through other mechanisms—remains a hypothesis to be tested. Previous studies have reported that FGL2 exhibits procoagulant and immunosuppressive functions, primarily through its thrombin-like activity and interaction with Fcγ receptors, rather than through direct complement C3 binding or activation. In contrast, our study identifies a direct protein-protein interaction between FGL2 and C3 in AKI, suggesting a distinct, context-dependent mechanism. Future investigations should directly assess the effect of FGL2 on C3 convertase activity in cell-free or reconstituted systems, or examine whether FGL2 interacts with complement regulatory proteins to modulate C3 activation. Third, although the cellular source of pathogenic FGL2 in AKI has now been identified, the possibility of a minor contribution from infiltrating immune cells cannot be completely excluded. Our data pinpoint renal tubular epithelial cells as the dominant source. Furthermore, considering the well-established immunosuppressive role of Treg-derived FGL2 in autoimmune diseases and tumors [37,38], it is plausible that the overall effect of FGL2 in AKI reflects a balance between its pro-inflammatory and potential immunosuppressive functions. Future studies employing cell type–specific knockout models will be essential to dissect these opposing roles. Fourth, the precise structural domains and molecular interfaces mediating the FGL2-C3 interaction remain unclear. Elucidating these mechanistic details is essential for the future development of targeted inhibitors. Fifth, this study has limitations including a small sample size (*n* = 12) and the lack of randomization or blinding in the animal experiments. Future studies will follow the ARRIVE guidelines to improve experimental design and will conduct large-scale cohort studies to validate the translational value of FGL2 and C3a.

## Conclusion

This study unveiled the role of FGL2 in tubular inflammation during AKI through its interaction with C3. It not only provides a novel theoretical framework for understanding the inflammatory mechanisms underlying AKI but also lays foundation for the development of targeted therapeutic strategies.

## Supplementary Material

supplementary figure.docx

supplementary method.docx

supplementary table.docx

## Data Availability

The analysis conducted in this study is based on publicly available datasets from the GEO database, with the following accession information: Dataset A: “Tubular epithelial NF-κB activity regulates acute ischemic kidney injury”, Accession Number [GSE87024]. Dataset B: “Blockade of TNF-α after ischemia reperfusion injury ameliorates renal prognosis”, Accession Number [GSE39766]. All other data generated during the course of this research have been included as supplementary files accompanying this article. Additional information is available from the corresponding author upon reasonable request.
