# Supplementary material for: Fibrinogen-like protein 2-complement C3 interaction exacerbates tubular inflammation in acute kidney injury by elevating complement C3a levels
Source: Ren Fail. 2026 Jul 20;48(1):2701621. doi: 10.1080/0886022X.2026.2701621 (PMC13386581; doi:10.1080/0886022X.2026.2701621)
Supplement: supplementary figure.docx [file IRNF_A_2701621_SM8538.docx]

**Supplementary Figure**

**
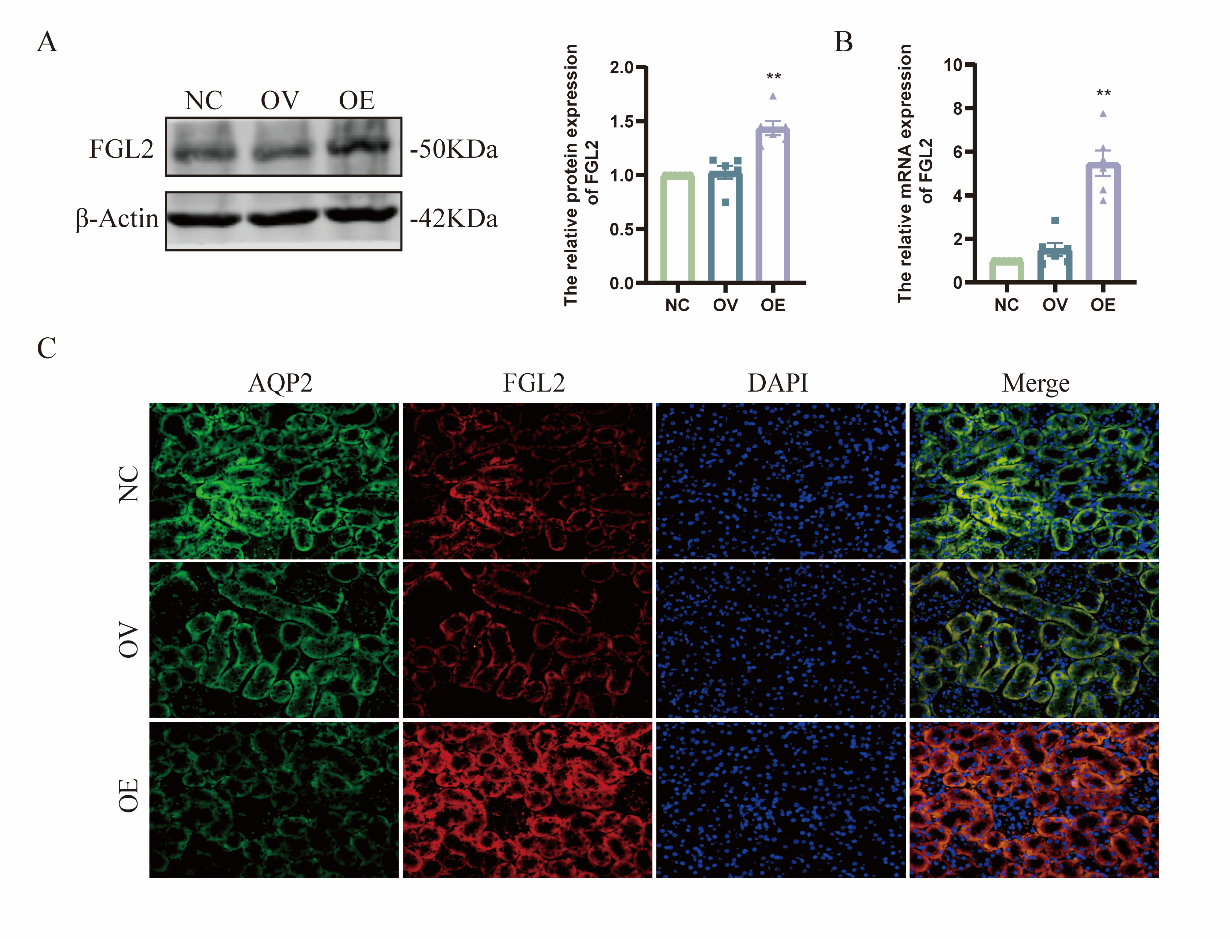
**

**Figure S1. Establishment of a Mouse Model with FGL2 Overexpression.** (A) Statistical results of FGL2 protein expression in the kidneys of mice across different groups, n = 6; (B) Statistical results of FGL2 mRNA expression in the kidneys of mice across different groups, n = 6; (C) Immunofluorescence images of FGL2 (red) and AQP2 (green) in renal tissues from mice in each group. Scale bar = 20 μm. Note: the normal control group (NC); the empty vector group (OV); the FGL2 overexpression group (OE). Data were represented as Mean ± SEM. ^*^*P* < 0.05; ^**^*P* < 0.01, compared with the NC group.


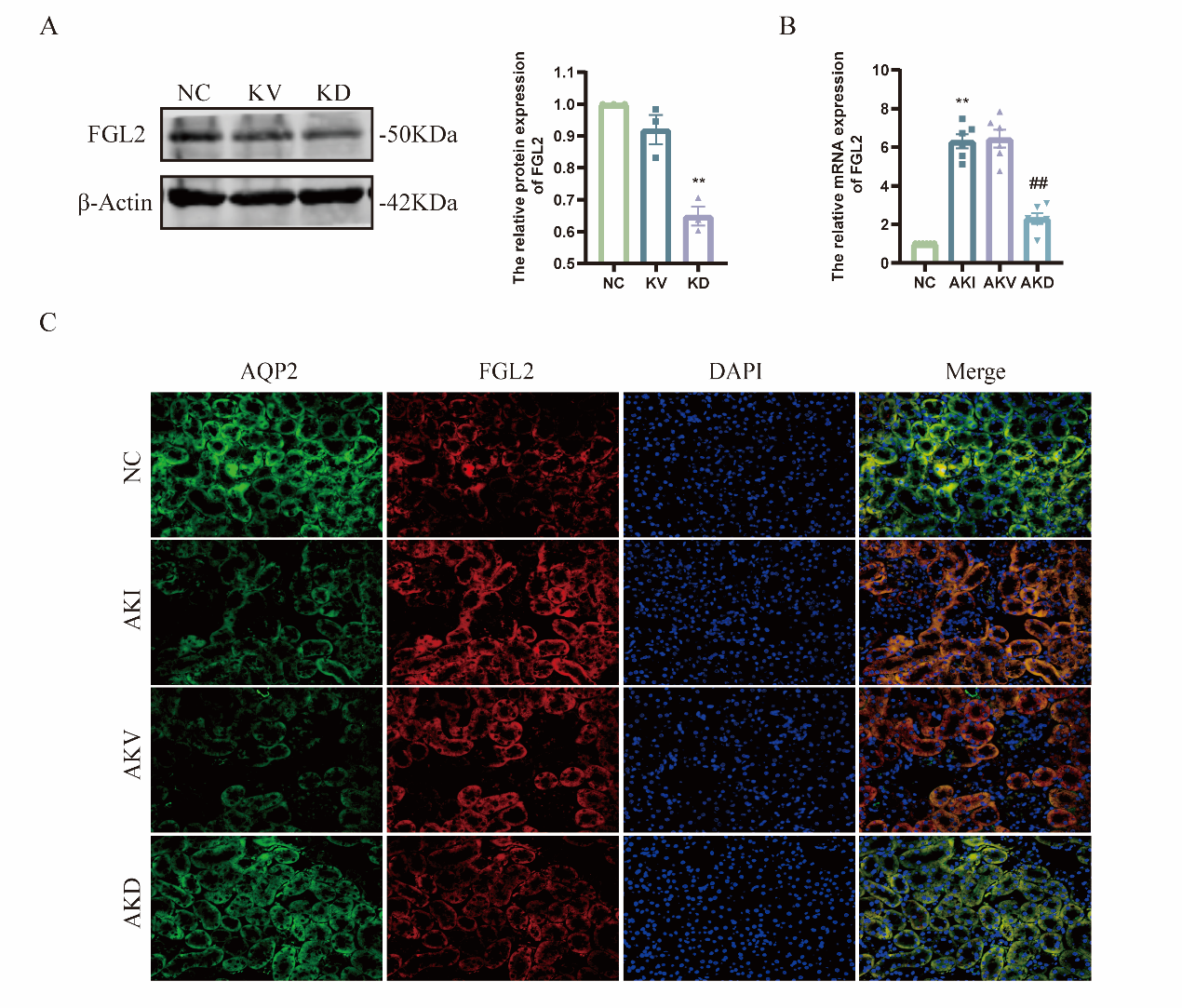


**Figure S2. Establishment of a Mouse Model with FGL2 Knockdown.** (A) Statistical results of FGL2 protein expression in the kidneys of mice across different groups, n = 3; (B) Statistical results of FGL2 mRNA expression in the kidneys of mice across different groups, n = 6; (C) Immunofluorescence images of FGL2 (red) and AQP2 (green) in renal tissues from mice in each group. Scale bar = 20 μm. Note: the normal control group (NC); the AKI group (AKI); the AKI + empty vector group (AKV); and the AKI + FGL2 knockdown group (AKD). Data were represented as Mean ± SEM. ^*^*P* < 0.05; ^**^*P* < 0.01, compared with the NC group. ^#^*P* < 0.05, ^##^*P* < 0.01, compared with the AKI group.


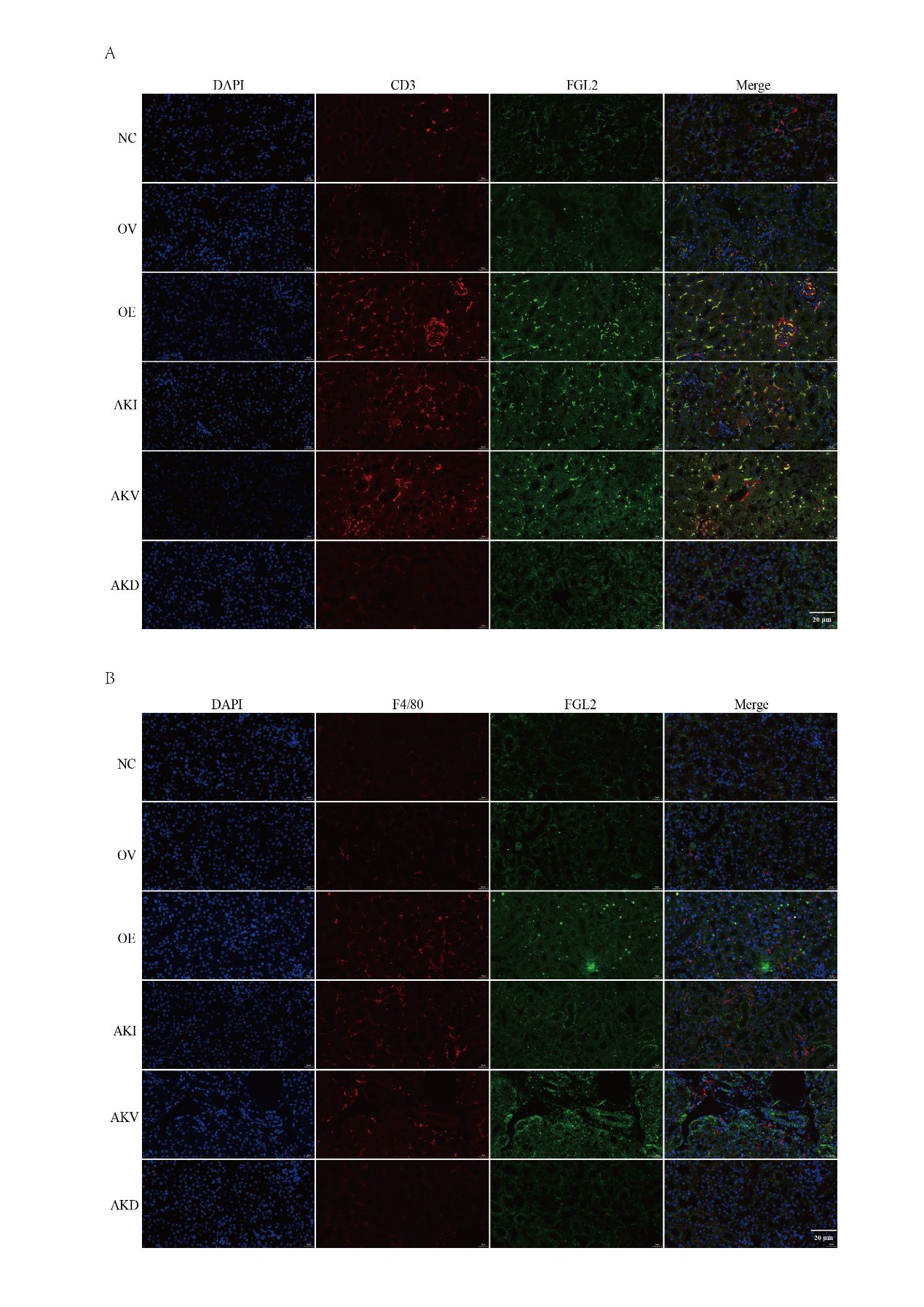


**Figure S3. Immunofluorescence co-staining shows no colocalization of FGL2 with CD3 or F4/80.** (A) Immunofluorescence images of CD3 (red) and FGL2 (green) in renal tissues from mice in each group. Scale bar = 20 μm; (B) Immunofluorescence images of F4/80 (red) and FGL2 (green) in renal tissues from mice in each group. Scale bar = 20 μm. Note: the normal control group (NC); the empty vector group (OV); the FGL2 overexpression group (OE); the AKI group (AKI); the AKI + empty vector group (AKV); and the AKI + FGL2 knockdown group (AKD).


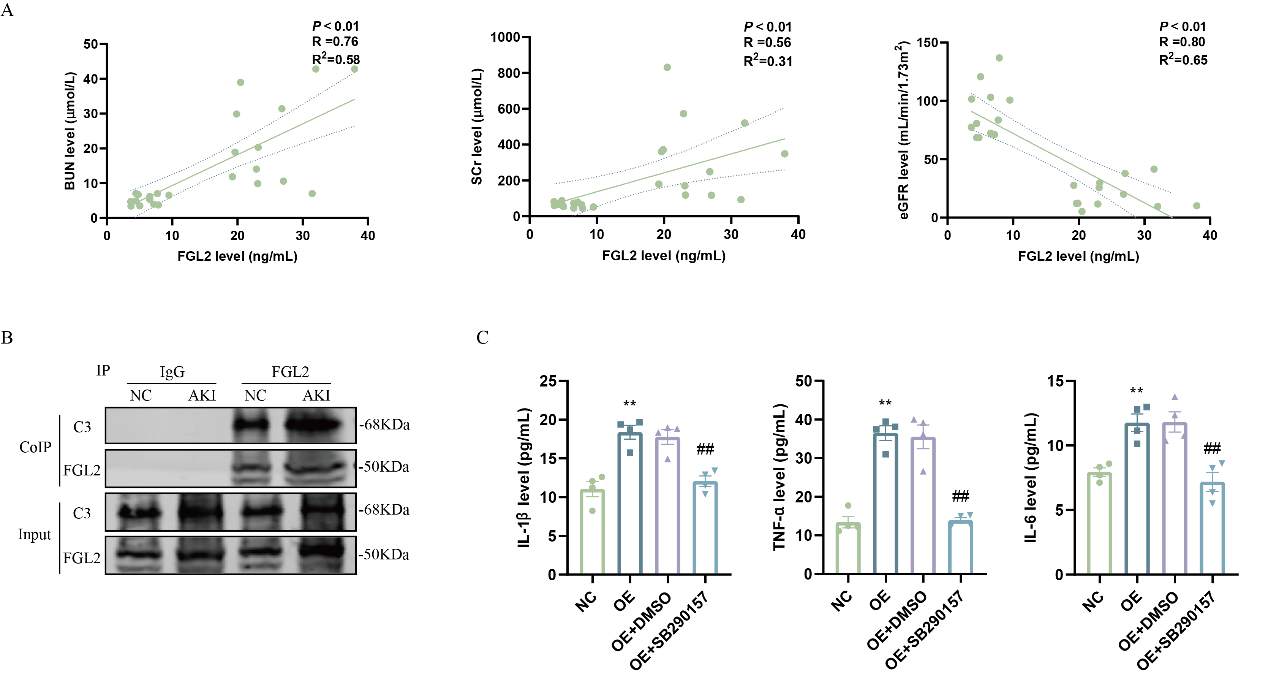


**Figure S4. Correlation of FGL2 expression with clinical parameters, Co-IP analysis of FGL2-C3 in mouse kidney tissue, and effects of antagonist treatment in HK-2 cells.** (A) Correlation between serum FGL2 expression levels and clinicopathological parameters. (B) Interaction between FGL2 and C3 in mouse kidney tissue. (C) Levels of inflammatory cytokines (IL-1β, TNF-α, IL-6) in each group of cells. Note: the normal control group (NC); the FGL2 overexpression group (OE); the OE + DMSO group (OE + DMSO); and the OE + SB290157 group (OE + SB290157). Data were represented as Mean ± SEM. ^*^*P* < 0.05; ^**^*P* < 0.01, compared with the NC group. ^#^*P* < 0.05, ^##^*P* < 0.01, compared with the OE group.
