# Supplementary material for: Fibrinogen-like protein 2-complement C3 interaction exacerbates tubular inflammation in acute kidney injury by elevating complement C3a levels
Source: Ren Fail. 2026 Jul 20;48(1):2701621. doi: 10.1080/0886022X.2026.2701621 (PMC13386581; doi:10.1080/0886022X.2026.2701621)
Supplement: supplementary method.docx [file IRNF_A_2701621_SM8537.docx]

**Clinical research.** Inclusion Criteria, the Normal Control (NC) Group: Aged 18 to 85 years; No abnormalities detected on renal pathological examination and no history of renal disease; Negative urine protein test, with creatinine and urea levels within the normal range. The Acute Kidney Injury (AKI) Group: Aged 18 to 85 years; Meeting the clinical diagnostic criteria for AKI as outlined in the 2012 KDIGO guidelines; Possession of complete clinical records and renal pathological data. Exclusion Criteria, the NC Group: Confirmed diagnosis of any renal disease; Abnormal liver or renal function test results; Severely incomplete clinical data or specimens that do not meet testing requirements. The AKI Group: Coexisting primary or secondary renal diseases; Confirmed malignancy with an expected survival period of less than 6 months; Severely incomplete clinical data or specimens that do not meet testing requirements. The glomerular filtration rate was estimated using the Chronic Kidney Disease Epidemiology Collaboration equation.

**Cell culture.** Cells in logarithmic growth phase with 20-30% confluency were selected for lentiviral transduction. In the FGL2 overexpression experiments, three groups of cells were established: the normal control group (NC), the empty vector group (OV), and the FGL2 overexpression group (OE). For the FGL2 knockdown experiments, four groups were set up: the normal control group (NC), the AKI group (AKI), the AKI + empty vector group (AKV), and the AKI + FGL2 knockdown group (AKD). Cells were cultured to 70-80% confluence, then the medium was replaced with low-serum medium containing 0.5% FBS. The C3aR antagonist SB290157 (TargetMol, T12851) was added, and the cells were incubated at 37°C in a 5% CO₂ incubator. After incubation, the cell culture supernatant was collected for analysis of relevant indicators. The experimental groups were set as follows: the normal control group (NC); the FGL2 overexpression group (OE); the OE + DMSO group (OE + DMSO); and the OE + SB290157 group (OE + SB290157).

**Animal experiment.** Mice were randomly assigned to six groups: the normal control group (NC), the empty vector group (OV), the FGL2 overexpression group (OE), the acute kidney injury group (AKI), the AKI + empty vector group (AKV), and the AKI + FGL2 knockdown group (AKD). There are at least 6 mice in each group. Mice in the control group received no treatment. Mice in the empty vector, overexpression, and knockdown groups were administered adeno-associated virus (AAVs) carrying the corresponding empty vector, FGL2 overexpression or knockdown cassette via tail vein injection at a dose of 5 × 10¹¹ vector genomes (v.g.) per mouse. The AAVs promoting FGL2 overexpression (5'-TCATCTTCTGACCAAGAGTA-3') and the AAVs facilitating FGL2 knockdown (5'-GGACAGTAGAGTCCAGGAACT-3') were obtained from Genechem Biotechnology Co., Ltd (Shanghai, China). All viral injections were administered 4 weeks prior to the renal ischemia-reperfusion surgery to ensure the stable expression of the genetically manipulated elements. Four weeks after viral injection, mice from the NC group, the OV group, and the OE group were euthanized. Through WB and qPCR analyses, it was confirmed that both the expression levels of FGL2 in the kidneys of the OE group mice were significantly elevated compared to those in the NC group (Supplementary FigureS1 A, B). Immunofluorescence staining further revealed that the upregulation of FGL2 protein was primarily localized in the renal tubules (Supplementary FigureS1 C). To validate the in vivo efficacy of the knockdown virus, three mice were randomly euthanized in advance before the ischemia-reperfusion injury (IRI) surgery. WB analysis demonstrated that, compared to control mice, the expression level of FGL2 protein in the kidneys of mice in the FGL2 knockdown group was significantly downregulated (Supplementary FigureS2 A). Furthermore, kidney tissues from mice in each group were collected after surgery. qPCR results showed that, compared to the NC group, the expression of FGL2 in the kidneys of the AKI group was significantly upregulated; whereas, compared to the AKI group, the expression of this gene in the AKD group was effectively suppressed (Supplementary FigureS2 B). The immunofluorescence staining results were consistent with the aforementioned molecular changes, showing a marked increase in FGL2 expression in the renal tubules of the AKI group, while this upregulation trend was blocked in the AKD group (Supplementary FigureS2 C). The method for establishing IRI model is as follows: Mice were fasted for 12 hours prior to surgery with water deprivation. Anesthesia was induced with 3% isoflurane inhalation and maintained with 1% isoflurane (RWD, R150-22). The animals were secured in supine position, followed by skin preparation and sterilization. A midline cervical incision was made to sequentially dissect muscle and fascia layers, fully exposing bilateral kidneys and renal pedicles. Both renal arteries and veins were clamped using vascular clips for 30 minutes to induce ischemia, after which the clips were removed to restore renal perfusion. The incision was closed layer by layer with sterile sutures, followed by iodine disinfection. Postoperative analgesia was provided with meloxicam (4 mg/kg, s.c.). Blood and urine samples were collected from all groups after modeling for renal function analysis. Upon euthanasia, part of the kidney tissue was fixed in 4% paraformaldehyde (Biosharp, BL539A), and the remaining samples were stored at -80°C for subsequent biochemical and histological examinations. All operations complied with the guidelines from the International Association for the Study of Pain and the standard for laboratory animal use.

**Co-Immunoprecipitation.** Total proteins were extracted and quantified. For each sample, 500 μg of protein was transferred into a microcentrifuge tube and adjusted to a final volume of 500 μL with lysis buffer. Subsequently, 2 μL of corresponding antibody (1: 200) and 20 μL of 50% protein A/G agarose beads (Santa, sc-2003) were added to each tube. The tubes were sealed with parafilm and incubated overnight at 4°C with gentle rotation on an orbital shaker. The following day, samples were centrifuged at 7,000 × g for 1 minute at 4°C, and the supernatant was discarded. The agarose beads were resuspended in 1 mL lysis buffer, centrifuged under identical conditions, and the washing step was repeated five times. After washing, 20μL of 2 × loading buffer was added to the bead pellets, followed by denaturation at 100°C for 10 minutes in a metal bath. Subsequent procedures were performed according to standard Western blotting protocols.
