# Supplementary material for: Fibrinogen-like protein 2-complement C3 interaction exacerbates tubular inflammation in acute kidney injury by elevating complement C3a levels
Source: Ren Fail. 2026 Jul 20;48(1):2701621. doi: 10.1080/0886022X.2026.2701621 (PMC13386581; doi:10.1080/0886022X.2026.2701621)
Supplement: supplementary table.docx [file IRNF_A_2701621_SM8536.docx]

**Table S1. Analysis of Baseline Characteristics in Clinical Patients.**

| Groups | NC  (n = 12) | AKI  (n = 12) | *P* value |
| --- | --- | --- | --- |
| Age (year) | 54.58 ± 12.20 | 60.08 ± 14.07 | 0.32 |
| Gender (male/female) | 5/7 | 7/5 | 0.68 |
| BMI (kg/m^2^) | 26.05 ± 2.74 | 24.17 ± 1.64 | 0.06 |
| BUN (μmol/L） | 5.27 ± 1.41 | 23.22 ± 13.38^**^ | < 0.01 |
| Scr (μmol/L） | 65.21 ± 13.92 | 327.79 ± 223.77^**^ | < 0.01 |
| eGFR (ml/min/1.73m2) | 90.43 ± 22.20 | 20.33 ± 12.01^**^ | < 0.01 |

Note: Data are presented as mean ± standard error of the mean (Mean ± SEM). ^**^*P* < 0.01, compared with the NC group.

**Table S2. The primers used for quantitative real time PCR.**

| Genes | Forward primers (5’-3’) | Reverse primers (5’-3’) |
| --- | --- | --- |
| FGL2 (human) | GGGGTGAGCAGCCTGTAA | GGGCAGACATCCTTTGCTCT |
| FGL2 (mouse) | CTCTGGGGGCAGCCTTCTC | GGCACTTCAGATTCCACCCA |
| C3aR (human) | CCAACAAGCAGGGGCTCTTA | TCCGCTGCTCACCATATCAC |
| C3aR (mouse) | CTCCTTGGCTCACCTGATTCTCC | ATGGCGAAGGCGGTTCTCAC |
| IL-1β (human) | CTGTACCTGTCCTGCGTGTT | GGGAACTGGGCAGACTCAAA |
| IL-1β (mouse) | GTCGCTCAGGGTCACAAGAA | GTGCTGCCTAATGTCCCCTT |
| C3  (mouse) | TGCTGGCCTCTGGAGTAGAT | AGGCAGTCTTCTTCGGTGTG |
| β-actin (human) | CATGTACGTTGCTATCCAGGC | CTCCTTAATGTCACGCACGAT |
| β-actin (mouse) | GTGACGTTGACATCCGTAAAGA | GTAACAGTCCGCCTAGAAGCAC |

**Table S3. The** **antibodies used in western blotting, immunofluorescence, and co-immunoprecipitation experiments.**

| Reagent (Catalog Number) | Manufacturer |
| --- | --- |
| β-Actin Monoclonal antibody (60008-1-Ig) | proteintech |
| FGL2 Polyclonal antibody (11827-1-AP) | proteintech |
| KIM-1/HAVCR1 Recombinant antibody (83221-2-RR) | proteintech |
| NGAL Polyclonal antibody (30700-1-AP) | proteintech |
| TNF-α Polyclonal antibody (29652-1-AP) | proteintech |
| IL-6 Polyclonal antibody (21865-1-AP) | proteintech |
| Anti-C3 antibody (Ab181147) | abcam |
| AQP2 (E-2) (sc-515770) | santa |

**Table S4.** **Molecular docking predicts the interaction between Fibrinogen Like 2 and core genes.**

| Protein ID | Gene name | Interface area, A² | ΔiG kcal/mol |
| --- | --- | --- | --- |
| P01027 | C3 | 1483 | -11.7 |
| P01582 | Il1a | - | - |
| P30882 | Ccl5 | - | - |
| E9Q9A6 | Il20rb | - | - |
| P06804C3 | Tnf | - | - |

**Note:** Binding energy (ΔiG), in kcal/mol, is determined as ΔiG = G-complex − (G-receptor + G-ligand). More negative values indicate stronger binding affinity.
